# Natural Occurrence in Venomous Arthropods of Antimicrobial Peptides Active against Protozoan Parasites

**DOI:** 10.3390/toxins11100563

**Published:** 2019-09-25

**Authors:** Elias Ferreira Sabiá Júnior, Luis Felipe Santos Menezes, Israel Flor Silva de Araújo, Elisabeth Ferroni Schwartz

**Affiliations:** Departamento de Ciências Fisiológicas, Instituto de Ciências Biológicas, Universidade de Brasília, Brasília, DF 70910-900, Brazil; elias.fsabia@gmail.com (E.F.S.J.); luisfelipe_100@outlook.com (L.F.S.M.); israelfsaraujo@gmail.com (I.F.S.d.A.)

**Keywords:** antimicrobial peptide, venom, arthropod, malaria, Chagas disease, human African trypanosomiasis, leishmaniasis, toxoplasmosis

## Abstract

Arthropoda is a phylum of invertebrates that has undergone remarkable evolutionary radiation, with a wide range of venomous animals. Arthropod venom is a complex mixture of molecules and a source of new compounds, including antimicrobial peptides (AMPs). Most AMPs affect membrane integrity and produce lethal pores in microorganisms, including protozoan pathogens, whereas others act on internal targets or by modulation of the host immune system. Protozoan parasites cause some serious life-threatening diseases among millions of people worldwide, mostly affecting the poorest in developing tropical regions. Humans can be infected with protozoan parasites belonging to the genera *Trypanosoma*, *Leishmania*, *Plasmodium*, and *Toxoplasma*, responsible for Chagas disease, human African trypanosomiasis, leishmaniasis, malaria, and toxoplasmosis. There is not yet any cure or vaccine for these illnesses, and the current antiprotozoal chemotherapeutic compounds are inefficient and toxic and have been in clinical use for decades, which increases drug resistance. In this review, we will present an overview of AMPs, the diverse modes of action of AMPs on protozoan targets, and the prospection of novel AMPs isolated from venomous arthropods with the potential to become novel clinical agents to treat protozoan-borne diseases.

## 1. Introduction

Arthropoda is a phylum of invertebrate animals that have a rigid exoskeleton with several pairs of articulated appendages whose number varies according to the class [1]. It is a diverse and ancient group of invertebrate animals, which underwent spectacular evolutionary radiation [2], totaling more than 5 million different organisms, approximately 80% of all known species on Earth [3,4,5,6]. This vast radiation allowed the occupation of a broad range of ecological niches, with gigantic variations in their lifestyle and dietary preferences [7,8,9,10,11,12].

The colonization of new environments probably enforced novel evolutionary challenges and requirements, improving morphological, biochemical, and behavioral features, enabling the selection of a series of exceptional adaptations, making them one of the first animal groups adapted to occupying terrestrial habitats [13,14]. Alongside these adaptations, evolutionary pressures on genes allowed the development of a highly efficient and rare predatory tool, found in only a few arthropod taxa: venom. New specialized organs or even whole venom delivery systems were evolutionarily selected (adapted) to actively inoculate venom inside the body of their victim, such as fangs or stings [15,16], resulting in a drastic increase in fitness, predatory success, and predator deterrence.

Venom apparatus is responsible for production of toxins, their storage and delivery through injection into prey [14,17,18]. Venom usage is so important in the animal kingdom that it evolved independently at least 19 times in arthropods [19]. Based on this vast radiation, the venom injection apparatus can be found in different arthropod body parts: in the distal end of the body, in the antennae, in the palpal chelae, present as modified legs, but most commonly in an adaptation of mouth parts [14,19]. Besides, venom has more specialized functions, such as preservation of prey for feeding parasitic larvae and aiding extra-oral digestion of prey [19,20]. The venom of the vast majority of arthropods is a complex mixture of peptides, proteins, and enzymes with a rich diversity of biological activities. Other minor components can be found in salt, inorganic ions, carbohydrates, glucose, and amino acids. [21,22,23]. Besides these, acypolyamines, biogenic amines, serotonin, histamine, protease inhibitors, mucopolysaccharides, proteases, hyaluronidase, phospholipases, and phosphoesterases can be found in the venom of scorpions and spiders [23,24,25].

Intriguingly, venomous animals belonging to the arthropod group are found in three major classes: Insecta, Arachnida, and Chilopoda. Recently, venom was described within the crustacean subphylum, the only species of venomous predator reported so far, the remipede *Xibalbanus tulumensis* [26]. Within the Insecta class, six orders have venomous representative species: Hemiptera, Neuroptera, Hymenoptera, Diptera, Lepidoptera, and Coleoptera. Together, these orders possess about 925,000 described species. The best studied order of venomous insects is Hymenoptera, comprising around 117,000 different species [27]. Regarding the Arachnida and Chilopoda classes, to date, the number of spiders, scorpions and chilopods described reached approximately 48,300, 2400, and 3200, respectively [28,29,30]. It has recently been suggested that ticks should be referred to as venomous ectoparasites, due to the composition and function of their saliva, and the clear differences between proteins present in tick saliva and other non-venomous animals. Tick saliva contains features of other venomous animals, such as proteins capable of inducing paralysis, interfering with normal host physiological processes [31].

Indeed, several drugs come from research on venomous animals. Captopril, Exenatide, and Ziconotide are some examples of biomolecules that have become drugs for the management and treatment of hypertension, diabetes and chronic pain, respectively [32,33,34]. In this context, venomous animals are a source of new compounds, arousing great interest from the biotechnology and pharmaceutical industries, making them apposite leading candidates for the development of new drugs.

## 2. AMPs

The majority of multicellular organisms are constantly vulnerable to dangerous pathogens, through contact and exposure in the environment. For their survival, they have created various mechanisms in a host defense network to combat this invasion [35,36,37]. AMPs represent the first-line host defense mechanism in all invertebrates; they were evolutionarily preserved as an essential component from the innate immune system, remaining an ancient (archaic), but powerful weapon throughout those years [38,39]. AMPs are usually small molecules (~10–50 residues long), gene-encoded, cationic, and amphipathic, with a miscellaneous composition of amino acids [40,41,42,43]. Despite their vast structural diversity, most AMPs kill pathogens microorganisms similarly, through membrane damage, protecting the host from bacteria, viruses, fungi, and parasites [44,45,46].

After microbial infection or even by means of stimuli such as stress, AMPs are synthesized in the fatty body of insects and hemocytes of invertebrates and, consequently, released into the hemolymph to combat infection [47]. Some genes encoding these peptides are intronless, suggesting that they are early response genes, facilitating post transcriptional modification and expression, working as a rapidly induced response to pathogens [48]. Furthermore, arthropod venom is also a vast source of AMPs, and it has been suggested that the presence of these biomolecules in venom works both in protecting the venom gland against microorganisms and in assisting the action of other toxins [49,50]. About 3000 antimicrobial peptides were described and isolated from six kingdoms (bacteria, archaea, protists, fungi, plants, and from animals) in recent years [51,52,53,54]. Antibacterial, antifungal, and antiparasitic peptides derived from these natural sequences have showed broad-spectrum and enhanced activity against target microorganisms [55,56,57].

Despite the vast diversity of sequences and sources of AMPs, they can be classified, according to structural features, into three main groups—α-Helical, β-sheet, and extended/flexible peptides [58,59,60]. The α-helical is the most common AMP structure, abundantly found in the extracellular fluids of insects, frogs, mammals, and other vertebrates. These molecules are free of cysteine residues and usually unstructured in aqueous solution but adopt the helical conformation upon contact with membranes [60,61]. β-sheet peptides are a diverse group of molecules, containing six to eight cysteine residues, responsible for formation of two or more disulfide bonds that will stabilize the β-sheet structure. They also present a well-defined number of β-strands, amphipathically organized, with distinguishable hydrophobic and hydrophilic surfaces [62,63,64]. The last subgroup of AMPs includes peptides that are linear without cysteine residues and possesses a unique extended coil structure. These AMPs have been less characterized, but they contain a high proportion of proline, arginine, tryptophan, glycine, and histidine [63,65,66,67].

Currently, over 10 AMPs have entered clinical trials or started the pre-clinical development stages [68,69]. The natural lipopeptide antibiotic approved by the Food and Drug Administration in 2003, named Daptomycin, and the glycopeptide Vancomycin are some examples of AMPs routinely used to treat drug-resistant Gram-positive bacteria. They are labeled “last resort” antibiotics, used only when clinical and commonly used drugs are not sufficient to stop the infection [70,71,72,73]. Additional efforts are necessary to extend these findings in the path to drug development and to prospect further the antiparasitic potential of AMPs from animal venoms.

## 3. Differences between Plasma Membranes of Protozoan and Mammalian Cells

The plasma membrane of mammalian cells contains over one hundred different lipids, carrying little net charge and possessing an even lower outer membrane charge, mainly because of the most common non-polar lipid cholesterol and the four major phospholipids present in this structure: zwitterionic phospholipids enriched with phosphatidylcholine and sphingomyelin, phosphatidylethanolamine, and phosphatidylserine [74,75,76,77]. These phospholipids are distributed irregularly between the inner and outer membrane bilayers. Negatively charged lipids are mostly confined to the inner leaflet of the mammalian cytoplasmatic membrane, and the charges are not exposed, which could explain why AMPs do not target mammalian cells. Added to this, possible electrostatic interaction between AMPs and mammalian membrane cells is not stable and, if it happens, it will not affect the integrity of the lipidic bilayer [62,76,78,79].

On the other hand, the surface of the protozoan membrane is very conserved among individuals of this group, including the presence of glycosylphosphatidylinositol (GPI)-anchored glycoproteins, a covering that surrounds the cell membranes and forms the glycocalyx, a boundary between the parasite and the external environment, which also helps to form a negative net charge membrane. The glycocalyx mediates cell attachment, protects against harmful molecular and cellular agents, like AMPs, preventing their action on the membrane and/or affecting other vital functions [80,81].

In *Leishmania*, some free GPIs are also phosphoglycosylated to form lipophosphoglycan, the most common surface glycoconjugate of promastigote forms and a highly anionic GPI anchored component that, together with ergosterol, constitutes the principal molecules responsible for the negatively charged membrane of this parasite. Enzymes such as the metalloprotease Gp63 decrease the charge of the membrane, displaying a protective effect against AMPs through peptide cleavage, and are found in all developmental forms of the parasite, especially the promastigote form [82,83,84,85,86,87]. The toxicity of bombinin H2 and H4 peptides when tested against *Leishmania* promastigotes was considerably higher than treated amastigotes. These contrasting results are probably connected with the differences in glycocalyx complexity of these two different developmental forms. Intracellular amastigotes present an elementary organization, where glycocalyx is almost nonexistent, surrounded only by an endocytic vacuole of the phagocyte cell [70,88,89,90,91].

The glycocalyx surface of *T. cruzi* is mostly covered by mucin-like glycoproteins attached by GPI-anchored proteins. Free GPIs aggregate to form a densely filled glycocalyx beneath the mucin cover. The trans-sialidase family of glycoproteins is another molecule found in the cell surface of *T. cruzi*, playing a pivotal role in escaping from host immune surveillance [84,92,93,94,95].

*T. brucei* membrane surface coats are composed mainly of the variant surface glycoproteins (VSG) and are anchored to the outer membrane by a GPI-anchor [84]. *T. brucei* is an extracellular parasite in all developmental forms; consequently, these surface molecules are not used in cell attachment [96], but the VSG layer acts as a molecular sieve for particles over 20 kDa [97], besides protecting it from host complement via the alternative pathway. The parasite avoids the immune system thanks to its ability to express different VSGs and replaces them periodically, a phenomenon known as antigenic variation, allowing that *T. brucei* trypomastigotes persist for long periods in the human bloodstream [98,99].

During the intracellular life stage, *P. falciparum*–infected red blood cells (PfRBC) diverge from healthy red blood cells (RBC), mainly by an increase in phosphatidylinositol and phosphatidic acid and a decrease in sphingomyelin in the outer membrane [100]. These changes in RBC glycocalyx seem to be related to an electrostatic change in the outer membrane of PfRBC, explaining in part why cationic AMPs preferentially interact with cationic PfRBC glycocalyx and barely affect healthy RBC [101,102].

Tachyzoites is the motile, fast-growing, and intracellular stage of *T. gondii*. During this developmental form, it expresses a huge amount of GPI and free GPI in its glycocalyx. The free GPI has a glucose α1-4GalNAcβ1-4 disaccharide side chain, and when released from the parasite, generates a high immune response, activating macrophages and inducing the production of IgM antibody by the human host [103,104].

## 4. Mode of Action of Antiprotozoal AMPs

Since protozoan membranes are composed basically of negatively charged lipids and AMPs are cationic and amphipathic, electrostatic interactions between membrane and peptide must be related in the disruption mechanism of the surface-membranes. Conventional AMPs most likely target the cytoplasmic membrane, acting through permeabilization of the plasma membrane, disorganizing the electrochemical gradient, and consequently disrupting the cellular homeostasis of parasite cells [83]. Researchers believed that membrane targeting was AMPs’ only mode of action, but the mechanisms of action of these biotoxins have been considerably studied since their discovery [63]. Although investigations focus mainly on bacteria and fungi, targets and effects of AMPs against protozoa were elucidated, especially in *Leishmania* and trypanosomatidae parasites [61,70,105].

**Figure 1:**
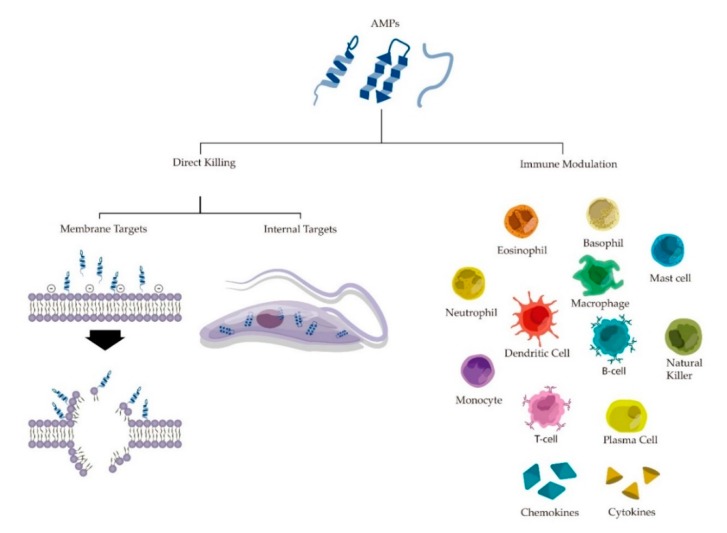
Mode of action of antiprotozoal AMPs. (**left**): Direct microbial action and possible membrane/internal targets of AMPs. (**right**): Modulation caused by AMPs in different types of cells, molecules and processes in mammals’ immune system.

The mode of action of AMPs can be divided into two major groups—direct microbial action in protozoan parasites (direct killing) and immune modulation of the host. In turn, direct action can be subdivided into AMPs targeting membrane and internal targets (Figure 1) [61,63,70,83,106,107].

### 4.1. Direct Killing

In the classical models of targeting membrane, the AMPs lying on the membrane must reach a critical concentration, capable of triggering the mechanism of membrane disarrangement. The interaction between the AMP and the parasite membrane does not involve receptor-specific interaction in most cases [108]. AMPs can have one or multiple microbial targets simultaneously, presenting a broad range of action against bacteria, viruses, parasites, and also anticancer activity [109]. Moreover, AMPs can show toxicity against different life cycles of the protozoa and sometimes even divergent mode of action for distinct developmental forms of the same organism [61].

Several models were suggested to explain the process induced by AMPs targeting membrane. The classical models of membrane disruption include the carpet model (detergent-like), the barrel-stave and the toroidal pore [70,83,110]. The carpet model proposes that electrostatic interactions cause peptide coating on the surface of the membrane and formation of a carpet structure, changing the fluidity and properties of the membrane, which will destabilize the bilayer through solubilization into micellar structures [108,111]. In the barrel-stave model, peptides self-aggregate and spontaneously insert themselves into the membrane, forming different sized pores, which grow in diameters according to the addition of new peptides [108,112]. The toroidal pore pattern shares common features with the barrel-stave, forming a membrane pore, but in this mechanism, peptides interact with the membrane, and transient pores are formed with peptides and lipids alternated in the arrangement. AMPs have been shown to translocate through the open pores, suggesting that this mechanism may be associated with potential intracellular targets [108,113]. Other modes of action models that try to describe targeting membrane were suggested, like molecular electroporation [110], sinking-raft model [114], Shai-Huang-Matsazuki model [115], the interfacial activity model [115], targeting of oxidized phospholipids [116], and anion carrier [117].

**Figure 2:**
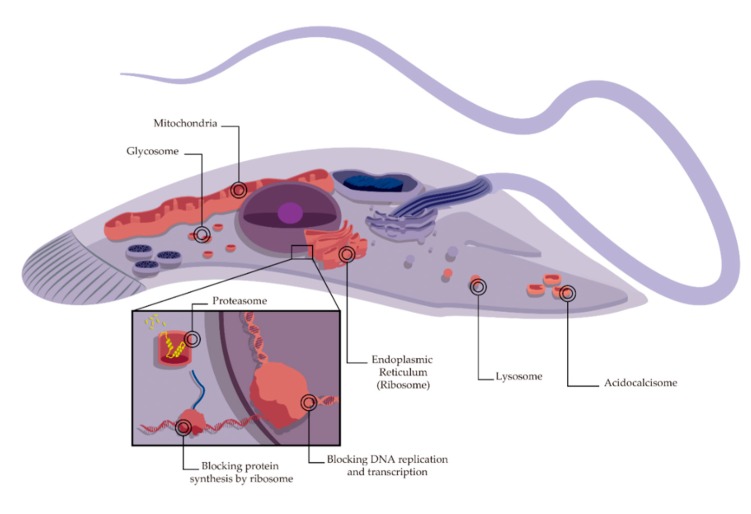
Schematic overview of a protozoan cell with various internal targets (highlighted in red) of AMPs.

Several internal targets were described for different parasiticidal AMPs, aiming at key cellular molecules and processes including DNA, RNA, and protein synthesis [118,119,120,121,122], protein degradation by proteasome [123], lysosomal bilayer [78], disrupting key enzymatic activities [124], organelles related with calcium storage (acidocalcisomes, glycosomes and/or endoplasmic reticulum) [125,126,127], and mitochondria (Figure 2) [79,128].

### 4.2. Immune Modulatory Effects

Several AMPs also are able to modulate the host immune system, displaying specificity toward a variety of immune responses: activation, chemotaxis, and differentiation of leukocytes, macrophage activation, degranulation of mast cells, changes in dendritic cell and adaptive immune responses, angiogenesis, cell proliferation, suppressing lactic acid formation, wound healing, controlling reactive oxygen, and nitrogen compounds and repressing inflammation through down-regulation of proinflammatory chemokines and pathogen antigens [107,129,130,131,132,133,134,135,136,137,138,139]. Generally, studies involving immune modulation of mammals by AMPs are done with bacteria. However, in view of some similarities in immune system responses against microorganisms, mammals’ immune modulation against protozoan parasites may present great similarities or in some cases even be identical to the bacterial model [140].

Most AMPs act through upregulation and activation of human immune system; however some AMPs work in a totally opposite way, inhibiting the inflammatory response through suppression of pro-inflammatory cytokines [132,140,141]. Innate defense regulators (IDR) are synthetic versions of natural AMPs, like IDR-1018. These peptides could be potential new drugs for treatment of severe malaria, since they decrease the harmful neural inflammation caused by *Plasmodium* infection, which is related to malaria patients’ mortality [132,141]. Phospholipase A2 from *Bothrops marajoensis* and *Apis mellifera* venom has shown antiparasiticial and immunomodulatory activities on *L. infantum*, *T. cruzi*, T. *brucei*, and *P. falciparum*. Besides that, temporins, magainin 2, and indolicidin can improve the efficiency of these venom enzymes through modulation of hydrolytic activity [137,142,143,144,145]. Because of that, some AMPs such as temporin and IDR-1018 may act as adjuvants, improving the effects and acting synergistically with other molecules, including AMPs [146].

## 5. Protozoonosis

### 5.1. Chagas Disease

**Figure 3:**
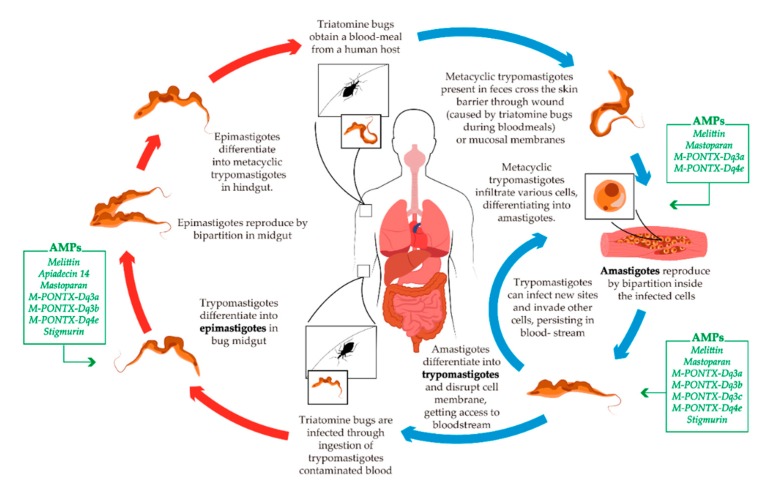
Schematic life cycle of *T. cruzi*. The blue arrows indicate life stages in the definitive host (human). The red arrows indicate life stages in the vector of CD. The green boxes illustrate the AMPs described with activity against each specific developmental form of the parasite.

*T. cruzi* is a parasitic protozoan and the causative agent of American trypanosomiasis, also known as Chagas disease (CD). CD is a vector-borne illness transmitted to animals and people predominantly by blood-sucking bugs (kissing bugs), mediated via infected insect’s feces, released during blood meals (Figure 3) [147,148,149]. The most important insects responsible for transmission of *T. cruzi* are members of *Rhodnius*, *Triatoma*, and *Panstrongylus* genera, which belong to the Triatominae subfamily [150]. Only two drugs are currently used in the clinical treatment of CD—benznidazole and nifurtimox. In spite of the fact that they are highly toxic, and their efficacy profile is far from ideal, both medicaments have been the frontline treatment for *T. cruzi* for nearly 50 years. Although both drugs are classified as essential by the WHO, they are not yet registered in Europe [148,150,151]. The use of benznidazole is approved by the FDA, but the need of high administered doses, the long period of treatment, the high incidence of side effects and the marked adverse reactions are some problems reported in its use. On the other hand, Nifurtimox is not currently FDA-approved [148,152,153]. Alongside this, *T. cruzi* strains resistant to these drugs were reported [154]. Therefore, there is a great need for new and safe parasitic drugs, especially due to the lack of efficiency of the main drugs on the market.

#### Anti-Chagas diseaseAMPs

The AMPs that exhibited toxicity and anti-*Trypanosoma cruzi* activity are summarized in Table 1, and the activity of the listed AMPs on specific stages of the life cycles is highlighted in Figure 3.

Melittin is an AMP from the western honeybee, *A. mellifera*, and the most abundant compound found in this insect venom. It is a 26-residue highly hydrophobic peptide, with 2.85 kDa molecular weight, presenting a small hydrophilic C-terminus, due to the presence of lysine and arginine amino acids. These features suggest that the peptide exerts its initial action at the parasitic membrane, and thanks to its amphipathic nature, the α-helical peptide binds to the membrane, causing destabilization. Melittin-treated epimastigote and amastigote cells presented changes in growth, viability and morphology, suggesting a predominantly autophagic death pathway. In addition, melittin exerts a calcium influx and does not disrupt the membrane permeability of *T. cruzi* bloodstream form, possibly involving apoptosis-like cell death, through an electrogenic process in a receptor-independent way. These results show that the same compound can induce different cell death mechanisms. The hemolytic effect of melittin does not make it so attractive to the pharmaceutical industry, but the use of hybrid AMPs, such as the hybrid of cecropin/melittin, substantially lessens this unwanted effect [155,156,157,158,159].

Apidaecin 14 is another AMP isolated from western honeybee venom. This insect toxin is heat-stable, 18 residues long, with 2.1 kDa, belonging to the proline-rich family of apidaecins, and differently from melittin, it is not an α-helical peptide, but a linear peptide with C-terminal amidation. It was bioassayed against *T. cruzi* epimastigotes with an innovative approach. In 2010, Fieck and co-workers used paratransgenesis to control *T. cruzi* in the vector *Rhodnius prolixus*. For this, they heterologously expressed different AMPs, using the symbiont microorganism *Rhodococcus rhodnii*, present in the same niche as the *T. cruzi* parasite: the insect’s gut. Apidaecin 14 showed lethality to *T. cruzi* with low toxicity to *R. rhodnii*. Surprisingly, the synergistic treatment of apidaecin with other AMPs (cecropin, magainin 2, or melittin) demonstrated high efficiency with half maximal inhibitory concentration values on the nanomolar scale [160]. The mode of action of apidaecin 14 seems to be related to the interaction and inactivation of the heat shock protein DnaK, an essential chaperone in several cytoplasmic cellular processes, including folding of nascent polypeptide chains, avoiding aggregation of partially folded proteins, remodeling folding pathways, and regulating activity [161,162].

Mastoparan is 14 amino acids in length and amidated in the C-terminus, isolated from *Polybia paulista* wasp venom with a molecular weight of 1.66k Da. The peptide is rich in hydrophobic and basic residues, which enable the formation of the secondary α-helical structure of the peptide. Unlike other AMPs, mastoparan exerts its toxicity by a unique mechanism. It inhibits glyceraldehyde-3-phosphate dehydrogenase from *T. cruzi* (TcGAPDH), a key enzyme in the glycolytic pathway. In addition, this peptide is related to ROS induction and mitochondrial disruption in all *T. cruzi* morphological forms, leading the cells to energy collapse [124].

Four different biotoxins active against *T. cruzi* were isolated from the venom of the New World giant ant *D. quadriceps*: M-PONTX-Dq3a, M-PONTX-Dq3b, M-PONTX-Dq3c, and M-PONTX-Dq4e. M-PONTX-Dq3b (13-residue peptide) and M-PONTX-Dq3c (11-residue peptide) are fragments of M-PONTX-Dq3a (23-residue peptide), with molecular weights of 1.5 kDa, 1.32 kDa, and 2.56 kDa, respectively. M-PONTX-Dq4e is the longest dinoponeratoxin, with 30 amino acids in length and 3.35 kDa. The four toxins present amidation at their C-terminus by post-translation modifications and the α-helical secondary structure. Among these, M-PONTX-Dq3a represents the most promising peptide from *D. quadriceps*, since it inhibits all *T. cruzi* developmental forms, including intracellular amastigotes. M-PONTX-Dq3a toxin has a high molecular weight and net charge, when compared to other dinoponeratoxins. This could be correlated with the high susceptibility of trypomastigote against this peptide, since this developmental form shows overexpression of sialic acid and mucin glycoproteins, negatively charged components of the parasitic plasmatic membrane. Against epimastigotes, M-PONTX-Dq3a showed inhibition rates 45 times lower than benznidazole, the first-line treatment for CD. Biochemical and morphological evidences suggest necrosis as the major death pathway of this AMP. These results against the developmental forms of *T. cruzi* are in agreement with the WHO guidelines for prospection of new drugs [163,166].

The α-helical peptide stigmurin, isolated from venom of the scorpion *T. stigmurus*, showed high antiparasitic activity on trypomastigote and epimastigote forms. This cationic peptide is formed by 17 amino acid residues and has 1.79 kDa molecular weight, with low hemolytic activity. Total growth inhibition of trypomastigote was achieved with a concentration of 25 µM of the toxin. Bioassays against epimastigotes with the same peptide concentration were able to inhibit 90% of parasite growth. Interestingly, rational designed peptides (StigA6, StigA16, StigA25, and StigA31) with higher net charge, increase in α-helix percentage and hydrophobic moment were able to inhibit the parasites with lower concentrations, when compared to native stigmurin. The analog peptides StigA6 and StigA16 presented 100% growth inhibition with a tenfold smaller dose, showing that rational design could be a promising tool to obtain effective new drugs. Stigmurin and the analogue peptides probably cause parasite death through interaction and destabilization of the cell membrane [164,167].

### 5.2. Human African Trypanosomiasis

**Figure 4:**
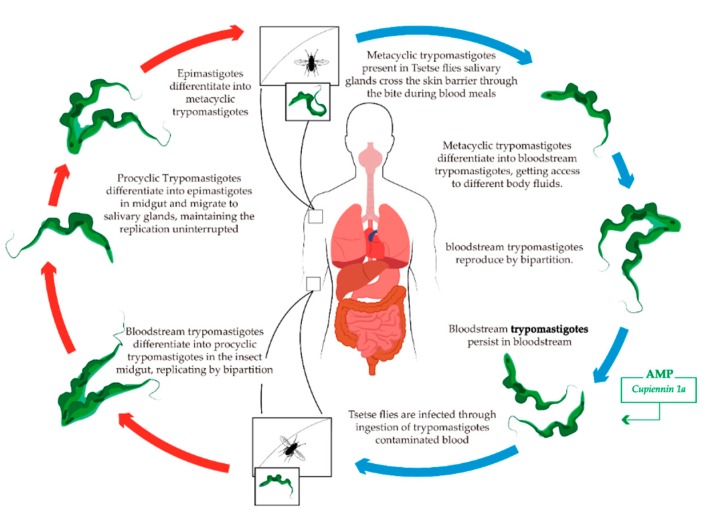
Schematic life cycle of *T. brucei*. The blue arrows indicate life stages in the definitive host (human). The red arrows indicate life stages in the vector of human African trypanosomiasis. The green box illustrates the AMP described with activity against the specific developmental form of the parasite.

*T. brucei* is a microscopic parasite and the disease-causing agent of Human African trypanosomiasis (HAT), also known as sleeping sickness, an illness spread via the bite of infected blood-feeding tsetse fly (genus *Glossina*) (Figure 4). Two different forms of the disease are known, depending of the subspecies of the parasite involved—West African trypanosomiasis (Gambian sleeping sickness) caused by *T. brucei gambiense* is responsible for the slow-progressing form. *T. brucei rhodesiense* is, in turn, behind the faster-progressing form, East African trypanosomiasis (Rhodesian sleeping sickness). Each subspecies of *T. brucei* is transmitted by different species or subspecies of *Glossina* [105,148,168]. Gambian sleeping sickness is doubtless the most common and widespread form of HAT, representing 98% of reported cases. In contrast, Rhodesian sleeping sickness is a zoonotic pathogen, affecting humans sporadically and responsible for only 2% of reported cases [168,169,170].

Sleeping sickness is curable with the right diagnostic approach and treatment but is lethal if untreated. The treatment in most cases needs much effort, mainly because of the logistic difficulties of drug delivery and access by professionals in rural areas for diagnosis and therapy administration.

The selection of therapy depends on both disease stage and the subspecies of the parasite. Currently, there are five first-line drugs routinely used against HAT. Pentamidine and suramin are used to treat first stage of Gambian and Rhodesian sleeping sickness, respectively. The second stage of *T. brucei gambiense* is treated with a combination of nifurtimox-eflornithine, which presents high trypanocidal efficiency, but the need for daily intravenous infusion and multiple administrations make this therapy regime difficult. For more than 70 years, the only treatment against East HAT has been Merlarsoprol, an arsenic-derived drug that presents many adverse reactions and highly toxicity, including encephalopathic reaction with mortality rate of approximately 10% of treated individuals. A new and revolutionary oral treatment, fexinidazole, was developed in 2018, and is able to cure both late and first stages of Gambian sleeping sickness. This pill-based therapy has received a positive scientific opinion from the European Medicines Agency and is already registered in the country with the highest incidence of cases, Congo [105,168,171].

#### Anti-Human African Trypanosomiasis AMPs

The only described AMP isolated from a venomous arthropod and active against *T. brucei* is the α-helical spider toxin Cupiennin 1a, a 35-residue cytolytic peptide isolated from the venom of the tiger wandering spider *Cupiennius salei* (Figure 4). This 3.5 kDa peptide exhibits broad activity against the parasites *T. brucei rhodesiense*, *T. cruzi*, and *P. falciparum*, with growth inhibition values at the nanomolar scale against *T. brucei rhodesiense* bloodstream forms. On the other hand, it also shows high cytolytic activity against negatively charged mammalian cells, mediated especially by sialic acid present in cell membranes, contributing to toxin-membrane interaction [165].

### 5.3. Leishmaniasis

**Figure 5:**
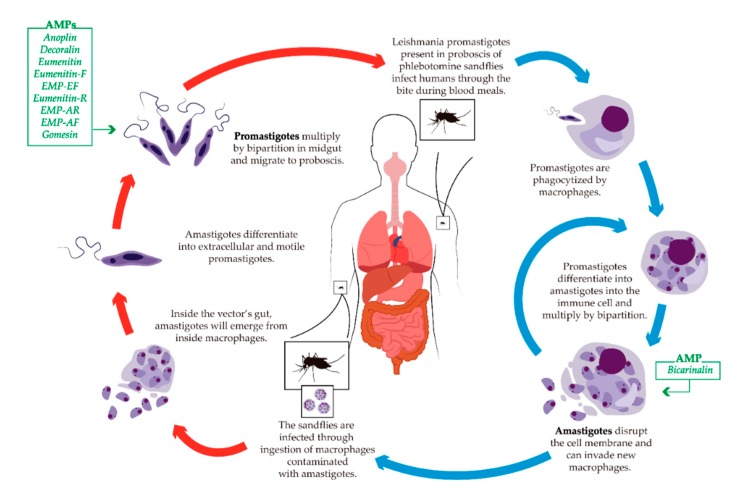
Schematic life cycle of *Leishmania*. The blue arrows indicate life stages in the definitive host (human). The red arrows indicate life stages in the vector of leishmaniasis. The green boxes illustrate the AMPs described with activity against each specific developmental form of the parasite.

Leishmaniasis is a vector-borne disease that is caused by obligate intracellular protozoan of the *Leishmania* genus [172,173]. Dipterans from the *Phlebotomus* genus are responsible for parasite transmission in the new world, while *Lutzomyia* genus causes transmission in the old world [173,174]. *Leishmania* are a complex group of unicellular parasites that alternately infect insects (intermediate host) and mammals (definitive host). About 70 different animal species are considered natural reservoir hosts of *Leishmania* parasites and more than 20 *Leishmania* species related to human infection [175,176]. There are several clinical presentation forms of leishmaniasis in humans. The three most common forms are cutaneous leishmaniasis (CL), visceral leishmaniasis (VL), and mucocutaneous leishmaniasis (MC). The *Leishmania* parasite, differently from other protozoan parasites, has a simple life cycle with only two digenic forms during the whole life cycle (Figure 5) [177,178].

Leishmanial treatment is conditioned by several factors, comprising type of disease, concomitant pathologies, parasite species and geographic location [173]. A huge number of drugs for the treatment of each leishmaniasis form are available, but pentavalent antimonials (stibogluconate and meglumine antimoniate) have been the first line and most used compounds in the treatment of all leishmaniasis forms for decades [179,180]. Pentavalent antimony administration is done parenterally for 28 days, making monitoring by health professionals necessary. In addition, these medicaments present high toxicity, adverse effects and an increase in parasite drug resistance [181,182,183]. For CL, other drugs like pentamidine and miltefosine are used, despite the excessive price, high toxicity and possible teratogenic side effects [181,184]. In India, miltefosine was used also to treat VL through oral administration [185], but a long treatment period increases the possibility of developing drug resistance [186]. The use of Amphotericin B has increased worldwide for VL treatment, but it causes significant nephrotoxicity [175,187,188].

#### Antileishmanial AMPs

The AMPs that exhibited toxicity and anti-*Leishmania* activity are summarized in Table 2, and the activity of the listed AMPs on specific stages of the life cycles is highlighted in Figure 5.

Gomesin was the first AMP isolated from a spider with toxicity against protozoan parasites. This defensin-type peptide was isolated from *Acanthoscurria gomesiana* hemocytes, possessing 18 amino acid residues and 2.27 kDa with four cysteine residues that form two internal disulfide bridges, contributing to stability and also responsible for the β-sheet structure of the peptide. Gomesin causes in vitro inhibition growth of *L. amazonensis* and *L. major* promastigotes at micromolar concentrations, which could be related to the high presence of anionic phospholipids and ergosterol in the plasma membrane of these parasites, causing a more negative net charge when compared with mammalian cells. This will allow the peptide to interact with the membrane, causing rupture and loss of cellular homeostasis [189,190].

Solitary wasp venoms can be a rich source of linear cationic α-helical peptides, killing parasites through membrane targeting. Decoralin (1.25 kDa), isolated from the venom of the solitary eumenine wasp *Oreumene decorates*, together with anoplin (1.15 kDa) from *Anoplius samariensis*, were bioassayed against *L. major* promastigotes. These peptides present some structural similarity, although decoralin has a linear chain length of 11 amino acid residues, one more residue than anoplin. Both peptides exhibited inhibition of promastigotes, despite a slightly high peptide concentration, but their hemolytic effect was quite low. The native peptide decoralin was synthesized with a C-terminal amidation (decoralin-NH_2_), and the analogous peptide demonstrated a sixfold reduction in the peptide concentration to exert the same growth inhibition as native decoralin, with no changes in hemolysis, possibly because C-terminal amidation stabilizes the α-helical conformation. All these features make the use of these toxins advantageous for chemical structure modifications and the improvement of biological properties [191]. A similar study carried out by Rangel and co-workers isolated four new linear cationic α-helical insect toxins from another two species of solitary wasps: two mastoparan peptides were isolated from *Eumenes rubrofemoratus*, the toxins eumenitin-R and eumenine mastoparan-ER (EMP-ER), and other two from *E. fraterculus*, eumenitin-F and eumenine mastoparan-EF (EMP-EF). Additionally, other two previously reported peptides were tested against *L. major* promastigotes, eumenitin from *E. rubronotatus* [192], and eumenine mastoparan-AF (EMP-AF) from *Anterhynchium flavomarginatum micado* [193]. All these six peptides showed some physicochemical and biological similarities: antileishmanial activity, short linear length (14 to 15 amino acids long), small molecular weight (1.48 to 1.65 kDa), polycationic features, and α-helical configuration after electrostatic interaction with anionic membrane. Among these peptides, EMP-ER, EMP-EF, EMP-AF present a C-terminal amidation, which may explain why EMP-ER demonstrates greater inhibitory effect against the promastigote form of the parasite [194]. Melittin showed robust inhibitory activity against *L. major* promastigotes, despite also exhibiting toxic effects on human dendritic cells [195]. A hybrid synthetic peptide using part of the melittin sequence and Cecropin A exhibited an enhancement in leishmanicidal activity and a decrease in host immune cell toxicity [196].

Bicarinalin is a recently characterized α-helical peptide and the first isolated from ants’ venom (*Tetramorium bicarinatum*) with trypanocidal activity. This biotoxin is a cystein-free polycationic peptide, with amidated C-terminal, 20 residues in length and 2.21 kDa, presenting very low hemolytic activity against human erythrocytes. Bicarinalin showed a broad spectrum of antimicrobial activities, a relatively long half-life stability for blood proteases (about 15 h) and slight cytotoxicity on human lymphocytes; in vitro bioassays against *L. infantum* intracellular amastigotes indicated parasiticidal activity at low concentrations. Thus, the membrane targeting bicarinalin shows signs of being a possible candidate for the development of a new leishmanicidal drug [197].

Promising studies showed the crude venom of *Tityus discrepans*, a medically important Venezuelan scorpion, inhibited the growth of *L. mexicana*, *L. braziliensis*, and *L. chagasi* promastigote forms, leading to drastic morphological alterations and consequently parasite death [198]. A preliminary study with crude venom of *D. quadriceps* giant ant displayed inhibition of promastigote forms of *L. amazonensis*. Flow cytometry and confocal microscopy analyses suggested involvement of necrotic and apoptotic pathways [199]. Interestingly, hybrid AMPs present notable in vitro antileishmanial activity with enhanced activity on the parental peptides and less hemolytic effects, in both life forms of *Leishmania*, intracellular amastigotes and extracellular promastigotes, besides a broad spectrum against different varieties of *Leishmania* [159,196,200].

### 5.4. Malaria

**Figure 6:**
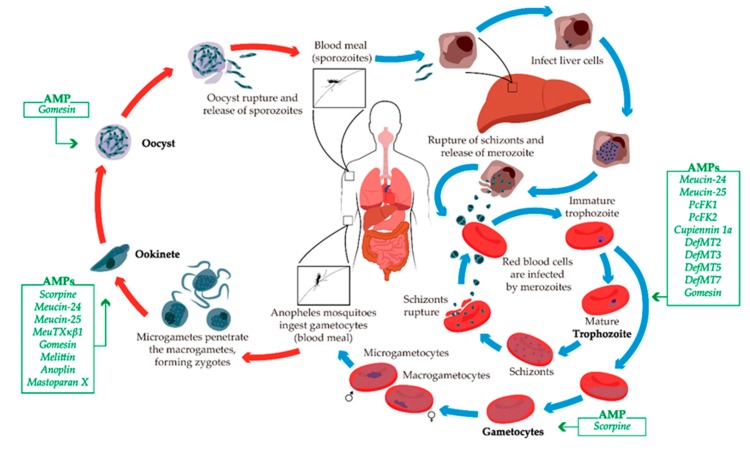
Schematic life cycle of *Plasmodium*. The blue arrows indicate life stages in the definitive host (human). The red arrows indicate life stages in the vector of malaria. The green boxes illustrate the AMPs described with activity against each specific developmental form of the parasite.

There are five possible protozoa that may be related to malaria, all belonging to the *Plasmodium* genus: *P. vivax*, *P. falciparum*, *P. malariae*, *P. ovale*, and *P. knowlesi* [201]. Transmission occurs through the bite of female mosquitoes of the genus *Anopheles* carrying the protozoan (Figure 6) [202]. The chosen treatment depends on the type of *Plasmodium*, the severity of the disease and the locality in which the disease was acquired [148]. This identification is important to determine the resistance probability of the organism to a particular drug. Among the antimalarial drugs used, the first-line drugs are chloroquine, atovaquone-lumefantrine (Malarone), artemether-lumefantrine (Coartem), doxycycline, primaquine, and tafenoquine [203,204,205].

#### Antimalarial AMPs

The AMPs that exhibited toxicity and anti-malaria activity are summarized in Table 3, and the activity of the listed AMPs on specific stages of the life cycles is highlighted in Figure 6.

Scorpine, an AMP from *Pandinus imperator* scorpion venom, has 75 amino acids in length, a molecular mass of 8.3 kDa and three disulfide bridges, and it presents anti-bacterial and anti-malarial activities. The results showed that the peptide was active in the sexual stages of the parasite. Scorpine inhibited ookinete and gamete development. When compared with shiva-3, a synthetic analog of cecropin peptide with antiparasitic activity, scorpine exhibited more potent toxicity in gametes and ookinetes than shiva-3 [206].

Meucin-24 and Meucin-25 are *Mesobuthus eupeus* scorpion venom AMPs, discovered through investigation of the cDNA venom gland library. Meucin-24 has 24 amino acids, 2.75 kDa and a high sequence identity with antimicrobial and K^+^-channel blocker toxins, also possessing N-terminus homology with melittin. Meucin-25 has 25 amino acids, 3.1 kDa, but no sequence identity with antimicrobial toxins described. They exhibited activity against *P. berghei*, *P. falciparum*, and also dipteran cells, making them potentially attractive for use with double action as a disease vector control tool and also as an antimalarial molecule. In water, meucin-24 showed a random coil conformation and meucin-25 a β-sheet structure. In TFE, both showed an α-helical formation [207].

MeuTXKβ1, a *Mesobuthus eupeus* venom toxin, did not show an effect on Na_v_ and K_v_ channels tested at concentration of 1 µM, and presented low antibacterial action, with a lethal concentration of 21 µM. However, activity against *P. berghei* development was stronger than the activity presented by other synthetic peptides, like shiva-3 [208]. In water, meuTXKβ1 showed 17% of α-Helix and 21% of β-sheet conformation and, in 50% of TFE, it showed 55% of α-Helix and 17% of β-sheets [209].

Psalmopeotoxin I and psalmopeotoxin II are AMPs isolated from *Psalmopoeus cambridgei*, the Trinidad chevron tarantula, also known as *Psalmopoeus cambridgei Falciparum* killer (PcFK). Both psalmopeotoxin I (PcFK1) and psalmopeotoxin II (PcFK2) peptides present three disulfide bridges, differing in the number and composition of amino acids in their structure. PcFK1 is a 33-residue peptide with 3.63 kDA, while PcFK2 is shorter, with a length of 28 amino acids and 2.96 kDa [210].

Gomesin showed activity against some bacteria and fungi and recently was compared to another five peptides with the same structure, in order to comprise the interconnection between the structural properties and the antimicrobial activity, deducing that high amphipathicity and low hydrophobicity of AMPs are related to more toxicity activity [211]. Moreira and co-workers tested gomesin against *P. berghei* and *P. falciparum*, besides analyzing the effect on the mosquito, aiming for antimalarial activity on the vector. The results showed an inhibition of gamete development and also of ookinete formation in *P. berghei*. In addition, the spider peptide displayed inhibition against the intraerythrocytic stage of *P. falciparum*. Gomesin manifested activity against oocysts in vivo for both parasite species, in the vector *A. stephensi*, and did not affect the mosquito’s development [212].

Cupiennin 1a displayed a non-stereospecific cytolytic activity against cancer cells, human blood, bacteria, trypanosomes and *Plasmodium*. This toxin was bioassayed against *P. falciparum* showing very low IC_50_ values, but a high hemolytic activity [165].

*I. ricinus* is a European tick that encodes antimicrobial peptides with action on pathogens such as bacteria and fungi. Cabezas-Cruz and co-workers studied the defensin peptides DefMT2, DefMT3, DefMT5, DefMT6 and DefMT7 against the malaria parasite. The results showed that the most effective peptide against *P. falciparum* was DefMT5. In contrast, DefMT6 did not show activity against *P. falciparum*, despite the similarity of these peptides. Regarding antibacterial and antifungal actions, DefMT3, DefMT5, and DefMT6 showed activity against both microorganisms, but DefMT2 and DefMT7 were not able to inhibit these pathogens [214]. All peptides have α-helix (N-terminus) and antiparallel β strand (C-terminus). Only DefMT7 does not present a β strand at the C-terminus [215].

Carter and co-workers tested several Hymenopteran AMPs that could show toxic effects on *Plasmodium* development (*P. berghei* and *P. falciparium*), namely melittin, anoplin, and mastoparan X isolated from *A. mellifera* [216], *A. samariensis* [217], and *V. lewisii* [218], respectively. Synergistic effects were also observed in treatments with two different peptides. Higher inhibition effect on the development of *Plasmodium* was observed when instead of using a single peptide (50 µM), two different peptides were administered together (25 µM each). For example, anoplin (25 µM) and mastoparan X (25 µM) showed a better inhibition effect than only mastoparan X (50 µM) [213].

### 5.5. Toxoplasmosis

**Figure 7:**
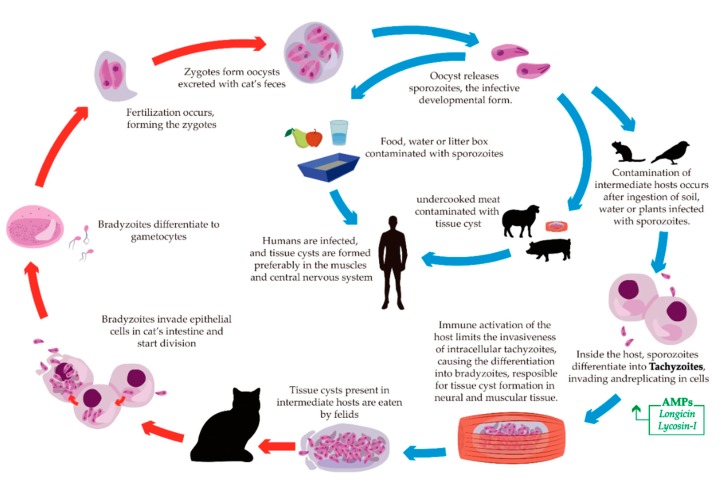
Schematic life cycle of *T. gondii*. The blue arrows indicate life stages in the intermediate hosts (exo-enteric cycle). The red arrows indicate life stages in the definitive host of toxoplasmosis (enteric cycle). The green box illustrates the AMPs described with activity against the specific developmental form of the parasite.

*T. gondii* is a protozoan that causes toxoplasmosis [219]. More than 40 million people worldwide have the parasite, although few have symptoms. Therefore, this disease is considered one of the neglected parasite infections (Figure 7) [148]. A few drugs for the treatment of toxoplasmosis are available, such as pyrimethamine and sulfadiazine [220], but studies using AMPs in this area are promising. So far, anti-*Toxoplasma* peptides were isolated from spider and tick, namely Lycosin-I [221] and Longicin [222], respectively.

#### Anti-*Toxoplasma* AMPs

The AMPs that exhibited toxicity and anti-toxoplasmosis activity are summarized in Table 4, and the activity of the listed AMPs on specific stages of the life cycles are highlighted in Figure 7.

Lycosin-I, from *L. singoriensis*, is a linear α-helical peptide with 24 amino acids and molecular weight of 2.89 kDa that inhibited *T. gondii* proliferation and invasion. The peptide was able to cause morphological changes in the parasite, causing damage to organelles, and vacuolization, signs of apoptosis-like death, but further studies are necessary to elucidate the death pathway caused by this spider toxin [221]. Longicin is a *H. longicornis* defensin peptide with β-sheet at the C terminus [223] that showed antibacterial and antiparasitic activities. The peptide precursor is formed by a 74-amino acids signal peptide, and the mature toxin is 52 residues in length, with 5.82 kDa. Tanaka and co-workers studied the peptide’s effect against *T. gondii* during the tachyzoite stage. The results showed morphological cell changes in cytoplasm and nuclei, consequently growth inhibition and parasite death, but the death pathway related to this peptide is still unclear [222].

## 6. Future Prospects

Several studies have been developed over the years, involving a wide variety of venomous animal AMPs tested against pathogenic protozoa, resulting in a bank with over 100 active molecules and potential agents for the development of novel peptide-based antiprotozoal chemotherapies [54]. Nevertheless, the need for new antiparasitic drugs is still urgent, making the prospection of new sources of bioactive molecules very attractive. Among the venomous arthropods, the centipedes (Chilopoda) comprise over 3000 species and are amongst the most remarkable sources of venom peptides. Several studies showed a significant antibacterial activity with over 30 AMPs isolated from these venomous animals; they are therefore a possible source of new compounds against protozoonosis [224,225].

Bioengineering tools to circumvent cytotoxicity and hemolysis problems, as well to enhance parasiticidal activity, were explored to overcome the drawbacks of therapeutic natural peptides. Structural analogs of natural AMPs and hybrid peptide formulations performed well in improving biological activity, including the analogs of stigmurin, stigA6, and stigA16, or CM11 and Oct-CA(1–7)M(2–9), melittin/cecropin A hybrids [164,167,196]. Moreover, C-terminal amidation of decoralin significantly decreased the values of IC_50_ when compared with the native peptide [191]. In order to make the net charge of the peptide more negative, amino acid substitutions could be another strategy to improve the biological activity. AMPs also demonstrated the potential for technological innovation due to synergistic interactions exhibited when used in combination with conventional antibiotics and other AMPs, drastically decreasing antimicrobial resistance [160,226].

The use of synthetic AMPs is still limited by the high production price when compared to conventional organic molecule drugs, and isolation from natural sources is not a viable solution. Studies have been carried out in the development of recombinant DNA methods to successfully synthesize and purify AMPs for cost-effective therapeutic application, but the commercial viability of these methods has yet to be evaluated [227,228,229]. In addition, since ribosome-synthesized AMPs are expressed by unique genes, they can be considered for use in gene therapy for introduction directly into infected tissue [61], possibly promoting a reduction in the cost associated with large-scale production and purification of AMPs. Application of new computational and experimental strategies aimed at downsizing, stabilization and other druggability issues are likely to reduce prices in the near future.

Nevertheless, it is known that a long test period is required before AMPs are available on the pharmaceutical market, as some adverse effects have yet to be overcome, such as hemolytic activity and cytotoxicity. Until now, no AMPs from venomous arthropods have become available for the treatment of parasitic diseases, but despite all the challenges involved in making AMPs a real treatment for protozoan diseases, at least six AMPs are currently undergoing clinical development in various therapeutic areas [63]. Pexiganan, the synthetic magainin analog, has reached phase III clinical trials. This arginine-rich variant peptide is capable of inducing apoptosis in *Leishmania* [230,231,232]. Clinical assays of the synthetic cecropin/melittin hybrid Oct-CA(1–7)M(2–9) were performed against naturally acquired leishmaniasis in dogs. The effectiveness of the peptide was confirmed with the cure of canine leishmaniasis after intravenous injection therapy, without observing side effects, even after six months of treatment [200].

## 7. Conclusions

Due to poor sanitation, difficult access to safe water, and scarcity of basic care policies, protozoan parasites still cause debilitating human diseases across the globe. In addition, there is a lack of interest from the pharmaceutical market in chemotherapy treatments, lack of research for more effective vaccines, and adverse effects of long-term parenteral treatments that cause toxicity in patients. Recently, the WHO brought to the public a new prevention weapon and hope in the fight against malaria, the first vaccine against *P. falciparum*. RTS, S/AS01 is the name of the vaccine that will provide partial protection against malaria in young children, especially in Africa, through routine immunization programs [233]. However, there is still a need for innovative treatments and tools to treat those who cannot benefit from this immunization. Compared to other drugs developed for chronic and noninfectious diseases, the use of protozoan-directed AMPs is still in its initial phase, although it indicates attractive pharmaceutical action to combat parasitic diseases [234]. Although their application is taking place very gradually, the new discoveries and research into medicinal peptides are proving to be a reality for the treatment of protozoonosis. It is hoped that this compilation will develop prospects for new strategies and paradigms in the application of AMPs, and AMP-based drugs should become a reality in upcoming years.

## Figures and Tables

**Table 1:** AMPs isolated from different venomous arthropods with activity against *T. cruzi*.

Source	AMP	Parasite Stage	Inhibition Activity ^a^	Reference
**Insect**				
*Apis mellifera*	Melittin	Epimastigote Trypomastigote Amastigote	IC_50_ = 2.44 μg/mL IC_50_ = 0.14 μg/mL IC_50_ = 0.22 μg/mL	[155]
*A. mellifera*	Apiadecin 14	Epimastigote	LD_100_ = 199 μM	[160]
*Polybia paulista*	Mastoparan	Epimastigote Trypomastigote Amastigote	IC_50_ = 61.4 μM IC_50_ = 5.31 μM ^b^	[124]
*Dinoponera quadriceps*	M-PONTX-Dq3a	Epimastigote Trypomastigote Amastigote	IC_50_ = 4.7 μM IC_50_ = 0.32 μM ^b^	[163]
*D. quadriceps*	M-PONTX-Dq3b	Epimastigote Trypomastigote	IC_50_ = 48.8 μM IC_50_ = 7.4 μM	[163]
*D. quadriceps*	M-PONTX-Dq3c	Trypomastigote	IC_50_ = 34.8 μM	[163]
*D. quadriceps*	M-PONTX-Dq4e	Epimastigote Trypomastigote Amastigote	IC_50_ = 23.5 μM IC_50_ = 4.7 μM ^b^	[163]
**Scorpion**				
*Tityus stigmurus*	Stigmurin	Epimastigote Trypomastigote	GI = 90% (25μM) GI = 100% (25μM)	[164]
**Spider**				
*Cupiennius salei*	Cupiennin 1a	Amastigote	IC_50_ = 0.92 μM	[165]

IC_50_: Half maximal inhibitory concentration. LD_100_: Absolute lethal dose. GI: growth inhibition. ^a^ 24 h of treatment. ^b^ Exhibited inhibition, but IC_50_ was not calculated.

**Table 2:** AMPs isolated from different venomous arthropods with activity against *Leishmania*.

Source	AMP	Activity against	Parasite Stage	Inhibition Activity ^a^	Reference
**Insect**					
*Apis mellifera*	Melittin	*L. major* *L. panamensis*	Promastigote	EC_50_ = 74.01 μg/mL EC_50_ ≥ 100 μg/mL	[195]
*Anoplius samariensis*	Anoplin	*L. major*	Promastigote	IC_50_ ≥ 87 μM	[191]
*Oreumenes decoratus*	Decoralin	*L. major*	Promastigote	IC_50_ = 72 μM	[191]
*Eumenes rubronotatus*	Eumenitin	*L. major*	Promastigote	IC_50_ = 35 μM	[191]
*Eumenes fraterculus*	Eumenitin-F	*L. major*	Promastigote	IC_50_ = 52 μM	[194]
*E. fraterculus*	Eumenine mastoparan-EF (EMP-EF)	*L. major*	Promastigote	IC_50_ = 40 μM	[194]
*E. rubrofemoratus*	eumenitin-R	*L. major*	Promastigote	IC_50_ ≥ 62 μM	[194]
*E. rubrofemoratus*	Eumenine mastoparan-ER (EMP-AR)	*L. major*	Promastigote	IC_50_ = 20 μM	[194]
*Anterhynchium flavomarginatum micado*	Eumenine mastoparan-AF (EMP-AF)	*L. major*	Promastigote	IC_50_ = 35 μM	[194]
*Tetramorium bicarinatum*	Bicarinalin	*L. infantum*	Amastigote	IC_50_ = 1.5 μM	[197]
**Spider**					
*Acanthoscurria gomesiana*	Gomesin *	*L. amazonensis* *L. major*	Promastigote	IC_50_ = ~5.0 μM IC_50_ = ~2.5 μM	[189,190]

* Peptides isolated from venomous animals, but not from venom glands. EC_50_: Half maximal effective concentration. IC_50_: Half maximal inhibitory concentration. ^a^ 24 h of treatment.

**Table 3:** AMPs isolated from different venomous arthropods with activity against *Plasmodium*.

Source	AMP	Activity against	Parasite Stage	Inhibition Activity	Reference
**Insect**					
*Apis mellifera*	Melittin	*P. berghei* *P. falciparum*	Ookinete	GI = 100% (50 µM) GI = 60% (50 µM)	[213]
*Anoplius samariensis*	Anoplin	*P. berghei*	Ookinete	GI = 100% (100 µM)	[213]
*Vespula lewisii*	Mastoparan X	*P. berghei* *P. falciparum*	Ookinete	GI = 100% (100 µM)	[213]
**Scorpion**					
*Pandinus imperator*	Scorpine	*P. berghei*	Gametocyte Ookinete	ED_50_ = 10 µM ED_50_ = 0.7 µM	[206]
*Mesobuthus eupeus*	Meucin-24	*P. berghei* *P. falciparum*	Ookinete Trophozoite	GI = 40% (20 µM) GI = 100% (10 µM)	[207]
*M. eupeus*	Meucin-25	*P. berghei* *P. falciparum*	Ookinete Trophozoite	GI = 50% (20 µM) GI = 100% (10 µM)	[207]
*M. eupeus*	MeuTXKβ1	*P. berghei*	Ookinete	GI = 89–98.8% (10-20 µM)	[209]
**Spider**					
*Psalmopoeus cambridgei*	Psalmopeotoxin I (PcFK1)	*P. falciparum*	Trophozoite	IC_50_^c^ = 1.59 µM	[210]
*P. cambridgei*	Psalmopeotoxin II (PcFK2)	*P. falciparum*	Trophozoite	IC_50_ = 1.15 µM	[210]
*Acanthoscurria gomesiana*	Gomesin *	*P. berghei* *P. falciparum*	Trophozoite Ookinete Oocysts	IC_50_ = 46.8 µM GI = 100% (50 µM) GI = 86% (100 µM)	[212]
*A. gomesiana*	Gomesin *	*P. falciparum*	Oocysts	GI = 100% (100 µM)	[212]
*Cupiennius salei*	Cupiennin 1a	*P. falciparum*	Trophozoite	IC_50_ = 0.032 µM	[165]
**Tick**					
*Ixodes ricinus*	DefMT2 *	*P. falciparum*	Trophozoite	GI = 70% (50 µM)	[214]
*I. ricinus*	DefMT3 *	*P. falciparum*	Trophozoite	GI = 50% (50 µM)	[214]
*I. ricinus*	DefMT5 *	*P. falciparum*	Trophozoite	GI = 100% (50 µM)	[214]
*I. ricinus*	DefMT7 *	*P. falciparum*	Trophozoite	GI = 30% (50 µM)	[214]

* Peptides isolated from venomous animals, but not from venom glands. GI: growth inhibition. ED_50_: Median effective dose. IC_50_: Half maximal inhibitory concentration.

**Table 4:** AMPs isolated from different venomous arthropods with activity against *T. gondii*.

Source	AMP	Activity Against	Parasite Stage	Inhibition Activity ^a^	Reference
**Spider**					
*Lycosa singoriensis*	Lycosin-I	*T. gondii*	Tachyzoite	IC_50_^b^ = 28 μM IC_50_^c^ = 10.08 μM	[221]
**Tick**					
*Haemaphysalis longicornis*	Longicin *	*T. gondii*	Tachyzoite	- ^d^	[222]

* Peptides isolated from venomous animals, but not from venom glands. ^a^ 24 h of treatment. ^b^ Inhibitory effects on proliferation of intracellular tachyzoites. ^c^ Inhibitory effects on invasion of parasite into host cells. ^d^ Exhibited inhibition, but IC_50_ was not calculated.
